# Antimicrobial peptides: a new class of antimalarial drugs?

**DOI:** 10.3389/fphar.2014.00275

**Published:** 2014-12-19

**Authors:** Nuno Vale, Luísa Aguiar, Paula Gomes

**Affiliations:** Department of Chemistry and Biochemistry, Faculty of Sciences, Centro de Investigação em Química, University of PortoPorto, Portugal

**Keywords:** AMP, amphipathic, antimalarial, antimicrobial, cationic, membranolytic, peptide, *Plasmodium spp.*

## Abstract

A range of antimicrobial peptides (AMP) exhibit activity on malaria parasites, *Plasmodium spp.*, in their blood or mosquito stages, or both. These peptides include a diverse array of both natural and synthetic molecules varying greatly in size, charge, hydrophobicity, and secondary structure features. Along with an overview of relevant literature reports regarding AMP that display antiplasmodial activity, this review makes a few considerations about those molecules as a potential new class of antimalarial drugs.

## INTRODUCTION

### NATURAL ANTIMICROBIAL PEPTIDES: SOLDIERS IN THE BODY’S FIRST LINE OF DEFENSE

Once the organism is invaded by a pathogen, a primary response from the host immune system comprises production of specific peptides as defense compounds. Several of these humoral response peptides exert antibacterial, antifungal, or antiviral properties (Bulet et al., 1999) and are known as host defense peptides, or antimicrobial peptides (AMP; Bell, 2011). Hence, AMP form the first line of host defense against infection and are a key component of the ancient innate immune system. Most AMP are small amphipathic peptides, usually with 15–45 amino acid (AA) residues, and, in general, are cationic at physiological pH (Boman, 2003).

Antimicrobial peptides, which may be encoded by separate genes or produced by non-ribosomal biosynthesis, have been identified in various species from bacteria to insects, amphibians to mammals, including humans (Zasloff, 2002; Pelegrini et al., 2011). In insects, AMP are synthesized in the fat body, in hemocytes, or epithelia, and are released into the hemolymph. In vertebrates, AMP are present in amphibian skin secretions (Simmaco et al., 1999) and epithelia (Ganz and Weiss, 1997; Bals et al., 1998); in mammals, AMP are also observed in lymphocytes (Agerberth et al., 2000) and leukocytes (Sorensen et al., 1997).

Because of their broad activity against microbes, and their expression triggered by various infections, AMP have been intensely examined as potential therapeutic agents (Zasloff, 2002). In 2004, the antimicrobial peptide database (APD, http://aps.unmc.edu/AP/main.php), created at the University of Nebraska Medical Center, already gathered a significant number of AMP that had been discovered at both the gene and protein levels (Wang and Wang, 2004). Later, APD has been updated and expanded to a second version that allows users to search peptides by families (e.g., bacteriocins, cyclotides, or defensins), sources (e.g., fish, frogs, or chicken), post-translational modifications (e.g., amidation, oxidation, lipidation, glycosylation, or inclusion of D-AA), and binding targets [e.g., cell membranes, proteins, nucleic acids, lipopolysaccharides (LPSs), or other sugars; Wang et al., 2009]. Today, there is a huge plethora of AMP of both natural and synthetic origin, as recently reviewed elsewhere (Som et al., 2008; Rotem and Mor, 2009; Kuroda and Gaputo, 2013; Pushpanathan et al., 2013; Sgolastra et al., 2013), highlighting AMP as relevant antibiotics (Fjell et al., 2011).

### ORGANIZING DIVERSITY: STRUCTURE-BASED CLASSIFICATION OF ANTIMICROBIAL PEPTIDES

The diversity of AMP reported since earlier disclosures in this area has soon made clear that some organization/classification of AMP families was needed. For instance, Boman (2003) proposed AMP to be split into three major groups: (a) linear α-helical peptides free of cysteine residues; (b) β-pleated peptides containing disulfide bridges; (c) peptides with an overrepresentation of certain AA, such as proline, arginine, tryptophan, or histidine. However, peptides that did not fit into any of these groups were later found to be antimicrobial, as is the case of circular peptides like θ-defensins (Lehrer et al., 2012) or cyclotides (Jagadish and Camarero, 2010). Hence, at present, four main types of AMP can be roughly distinguished:

#### α-helical peptides deprived of Cys residues

Linear cationic α-helical AMP are a class of small peptides whose charge is imparted by the presence of multiple Lys and Arg, but also with a substantial portion (50% or more) of hydrophobic residues. These peptides are known for their broad-spectrum antimicrobial activity and ability to modulate the innate immune response (Powers and Hancock, 2003). One example is that of melittin, an α-helical cationic peptide from the venom of *Apis mellifera* bees, composed of 26 AA residues and in which the amino-terminal region is predominantly hydrophobic whereas the carboxy-terminal region is hydrophilic due to the presence of a stretch of positively charged AA (Raghuraman and Chattopadhyay, 2007). Melittin is a potent antimicrobial that seems to promote membrane permeabilization through pore formation according to the toroidal model (Yang et al., 2001). However, its hemolytic activity is too high for clinical application as a selective AMP, which led to studies addressing synthesis and evaluation of the antimicrobial potential of hybrid peptide constructs where melittin (entire or partial AA sequence) was combined with other non-hemolytic AMP, such as cecropins (Boman et al., 1989; Bastos et al., 2008; López-Rojas et al., 2011). Cecropins constitute a well-known family of α-helical AMP that share a similar structure containing two α-helical domains linked by a flexible region. Insect cecropins are known to induce pore formation in negatively-charged bacterial membranes (Ekengren and Hultmark, 1999; Tanaka et al., 2008). In turn, a positive surface charge or cholesterol present in the membrane bilayer decreases the channel formation potency of cecropins (Christensen et al., 1988), which explains why these have little or no effect on eukaryotic cells (being non-hemolytic) that are richer in zwitterionic phospholipids and contain a high amount of cholesterol as compared to bacteria (Yeaman and Yount, 2003; Beevers and Dixon, 2010; Pretzel et al., 2013).

Other widely studied families of α-helical, linear and cysteine-free AMP are those of magainins and dermaseptins, both naturally occurring in amphibians. Magainins 1 and 2 adopt an α-helical conformation in solution (Zasloff, 1987), and have been proposed to induce toroidal pores in bacterial membranes (Ludtke et al., 1996). The non-hemolytic character of magainin 2 and its protocidal activity underlie its interest as a potential anti-parasitic agent, and also as a template for creation of more potent large spectrum AMP analogs, such as pexiganan (Ge et al., 1999). In what concerns peptides from the dermaseptin super-family, these exhibit a broad range of antimicrobial activity and some of them were found to aggregate on the bacterial membrane surface in a carpet-like manner (Pouny et al., 1992).

#### β-pleated peptides containing disulfide bridges

A classical example of this group of AMP is that of defensins, peptides mostly found in mammalian phagocytes that usually contain six Cys residues (eight Cys have been found in some insect defensins) stabilizing peptide structure by forming three intramolecular disulfide bridges (Selsted et al., 1985). The mechanism of action of these peptides seems to also involve pore formation inducing membrane permeabilization, which is more extensive on negatively charged phospholipid bilayers (Lehrer et al., 1989; Wimley et al., 1994).

#### Peptides rich in Pro, Gly, His, Arg, and Trp residues

This is a somewhat more heterogeneous group of AMP, as those included are diverse in sequence and tridimensional structure, sharing the feature of having an overrepresentation of certain AA, specifically, Pro, Gly, His, Arg, and Trp. From this follows that AMP of this group seem to also have diverse mechanisms of antimicrobial action, in some cases apparently involving intracellular targets (Otvos, 2005).

A family of Pro-rich AMP is that of apidaecins, short peptides that may adopt a polyproline type II helical structure which could be the structural basis to bind to specific targets underlying its antibacterial activity (Li et al., 2006). In fact, apidaecins do not seem to interact with microbial membranes through formation of pores, but rather by an energy-driven, eventually transporter-mediated, process (Castle et al., 1999).

Gly-rich AMP have been found with variable sizes and without any clear sequence signature, apart from the high proportion (25–50%) of glycine residues. These peptides are in general longer than AMP from other classes, have disordered structure in water, and tend to self-order when in contact with artificial membranes (Bruston et al., 2007). Attacins are family of six Gly-rich AMP that can be divided into four basic (A–D) and two acidic (E–F) peptides, probably derived from two attacin genes (Yi et al., 2014). Attacins inhibit the synthesis of outer membrane proteins of *Escherichia coli* by blocking transcription of the respective genes (Carlsson et al., 1991), which is presumably achieved by an indirect mechanism, since attacins bind to the bacterial LPS but do not need to enter the cell to exert their action (Carlsson et al., 1998).

Tryptophan-rich AMP contain more than 25% of this amino acid. In what concerns this class of AMP, the archetypical example is that of indolicin, which adopts no particular secondary structure in water, but seems to undergo significant structural changes in the vicinity of lipid bilayers, explaining its strong membrane affinity underlying its antimicrobial activity (Ladokhin and White, 2001). This peptide has the ability to permeate bacterial membranes and, depending of its tridimensional shape, inhibits DNA synthesis by binding to it (Hsu et al., 2005). Other examples of Trp-rich AMP include tritrpticin (Lawyer et al., 1996), lactoferricin B (Bellamy et al., 1992), and Pac-525 (Wei et al., 2006).

His-rich AMP usually have 25% of their AA content represented by His. In general, these peptides show a cationic amphipathic helical structure, and trigger microbial membrane disruption when adopting an alignment parallel to the membrane surface. Still, pore formation is not essential for the high antimicrobial activity of many His-rich AMP (Mason et al., 2009). Clavanin (van Kan et al., 2002), daptomycin (Jeu and Fung, 2004), LAH4 (Bechinger, 1996), or D-HALO-rev (Mason et al., 2009) are a few examples of this class of AMP.

#### Circular antimicrobial peptides

Discovery of antimicrobial activity on natural cyclic peptides that did not fit any of the previous three groups justifies the need to consider a fourth group, dedicated to circular AMP. θ-defensins, for instance, fit this group: they are cyclic octadecamers active against several Gram-positive and Gram-negative bacteria, fungi, and some viruses, which consist of a couple of antiparallel β-sheets linked by three disulfide bonds to produce a very stable structure (Lehrer et al., 2012). Some bacteriocins, which are polypeptide toxins produced by bacteria to inhibit the growth of competing bacterial species or strain(s) (Cotter et al., 2013), are also circular AMP; that is the case of AS-48, a cyclic 70-mer bacteriocin from *Enterococcus faecalis*, possessing an overall globular structure where five α-helices enclose a dense hydrophobic core (González et al., 2000). Finally, one of the most emblematic families of circular AMP is that of cyclotides (Jagadish and Camarero, 2010): these are plant-derived peptides, with approximately 30 AA, characterized by a head-to-tail cyclic backbone and three or four disulfide bonds forming the so-called cyclic cysteine knot (CCK; Craik et al., 1999), for which they are also known as “knotted peptides.” As a result of their singular structure, these peptides are extremely stable, retaining their biological activity after boiling and being extremely resistant to enzymatic degradation (Vila-Perelló and Andreu, 2005; Craik and Conibear, 2011).

## ANTIMICROBIAL PEPTIDES: A NEW SOLUTION AGAINST MALARIA?

### A MILLENARY WORLDWIDE DISEASE STILL FAR FROM ERADICATION

Human malaria is caused by any of five species of protozoal, apicomplexan parasites of the genus *Plasmodium*, *P. vivax*, *P. ovale*, *P. malariae*, *P. knowlesi,* and *P. falciparum*, the latter being the most virulent, the best characterized, and (along with *P. vivax*) the most widespread species. Rodent malaria parasites such as *P. berghei* and *P. yoelii*, the avian parasite *P. gallinaceum*, or the human parasite *P. falciparum* are the most well-studied and used to evaluate drug-parasite interactions (Prudêncio et al., 2006; Dixon et al., 2008; Mantel et al., 2013; Regev-Rudzki et al., 2013). Parasites of the *Apicomplexa* are important animal pathogens notable for their complex life cycles and highly specialized invasive forms. Besides *Plasmodia*, apicomplexan parasites include the agents of toxoplasmosis, cryptosporidiosis, and several other significant parasitic diseases (WHO, 2012; Singh and Daneshvar, 2013).

**FIGURE 1:**
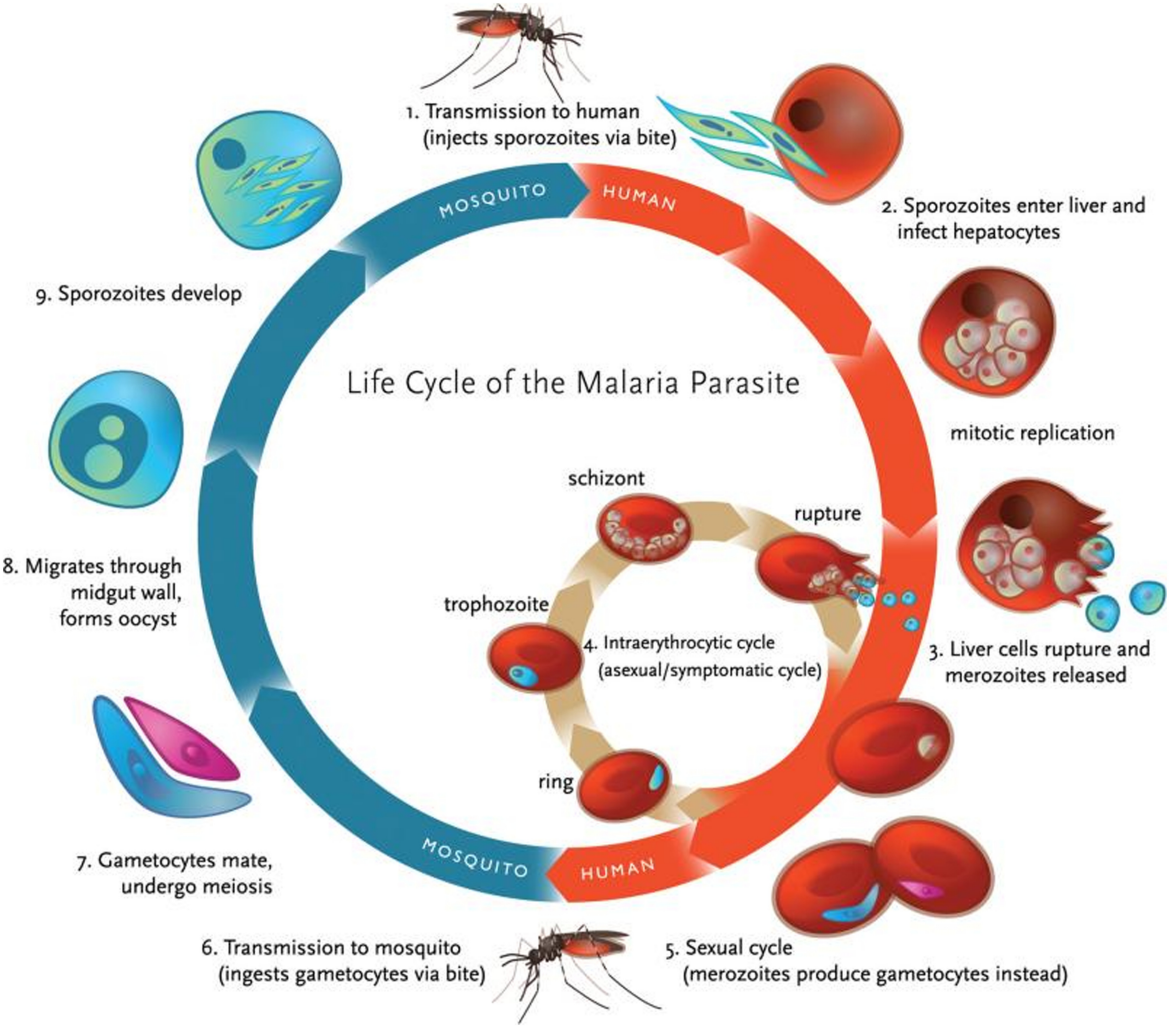
**Life cycle of malaria parasite.** Malaria transmission occurs through a vector, the female *Anopheles* mosquito, which ingests gametocytes (the only infective form to mosquitoes) when feeding on infected blood (adapted with permission from Klein, 2013).

The definitive host of *Plasmodia*, the female *Anopheles* mosquito, transmits the infective forms of the parasite, sporozoites, to the intermediate host (usually, a mammal) during its blood meal. After migration to the liver, the parasites develop within hepatocytes, of which they later exit as merozoites that are released into the bloodstream. Inside red blood cells (RBC) the asexual lifecycle takes place, starting by merozoite development into the ring stage, this in turn evolves to produce metabolically highly active trophozoites, which finally give place to schizonts, responsible for the release of new merozoites to infect other healthy RBC. Occasionally, ring forms can also develop into female and male gametocytes that, once ingested by another *Anopheles* mosquito, start the sexual lifecycle by developing into ookinetes, oocysts, and finally sporozoites, which migrate into the salivary gland to be transferred to another host on the following blood meal (**Figure 1**).

The complex life-cycle of malaria parasites, and the ease at which these undergo mutations to escape drug pressure, are the major factors behind both the limitations of current control methods and the urgent need for new chemoprophylactic and chemotherapeutic agents. These aspects have been comprehensively discussed elsewhere (Philips, 2001; Teixeira et al., 2014), turning clear that, while antimalarial drugs in use (mostly artemisinin and derivatives, quinolines or related compounds, and inhibitors of the folate pathway) can be effective in many situations, improvements in terms of (especially) cost and safety are highly desirable (Flannery et al., 2013; Visser et al., 2014; White et al., 2014). Yet, this is a difficult endeavor, as the mechanisms of action of many antimalarial drugs are still poorly understood. For instance, the current first-line antimalarial drugs, artemisinin and related compounds, have been suggested to eliminate the Ca^2+^-dependent ATPase activity of P*f*ATP6 (Eckstein-Ludwig et al., 2003); however, other modes of action have been attributed to this family of antimalarials as, e.g., binding to ferriprotoporphyrin IX (a by-product of hemoglobin degradation), free radical-mediated damage or interference with hematin polymerization and detoxification (Robert et al., 2002; Biagini et al., 2003). Other antimalarial drugs seem to target the redox systems of *Plasmodia*, whose survival is highly dependent on the antioxidative stress system of their hosts (Becker et al., 2004). An example is that of chloroquine, formerly used as first-line treatment for uncomplicated malaria, which inhibits polymerization of toxic heme (Fe^2+^) into hemozoin inside the parasite’s food vacuole (Sullivan et al., 1996). Primaquine, which remains the only transmission-blocking anti-malarial clinically available worldwide, seems to equally target the parasite’s redox system; this drug displays marked activity against gametocytes of all species of human malaria, including multi-resistant *P. falciparum* strains, and is also effective against all exoerythrocytic forms of the parasite, including hypnozoites, dormant liver forms responsible for relapse of *vivax* and *ovale* malaria. Unfortunately, as many other clinically relevant antimalarials, primaquine is often associated with serious adverse effects, in consequence of its toxic metabolites (Vale et al., 2009).

Another drawback in antimalarial containment has emerged from misuse of available drugs, and marketing of fake ones, leading to widespread resistance. This has been the major factor behind chloroquine’s loss of prominence in the antimalarial arsenal from the 1980s onward, and is also becoming a cause of concern regarding 21st century first-line artemisinin-based combination therapies (ACT): the first signs of plasmodial resistance to artemisinin emerged in Southeast Asia in 2008 (Dondorp et al., 2009).

The above explains why malaria eradication is still out of reach and why the need to feed the antimalarial drug pipeline remains an urgent problem. In this connection, membrane-active peptides (MAP), such as most AMP, may offer interesting solutions, as by having cell membranes as their primary targets, their action will be harder to fight back by malaria parasites.

### ANTIMICROBIAL PEPTIDES WITH ANTIMALARIAL PROPERTIES

There have been numerous reports on peptides active against various cultured stages of malaria parasites and/or in animal models of malaria. The range of size, AA composition, and secondary structure of such peptides is impressive, going from dipeptides up to polypeptides large enough to be considered as proteins. Antimalarial activity has been described for several substrate analogs of different plasmodial peptidases involved in host hemoglobin degradation, host-cell invasion and egress, and intracellular housekeeping (Blackman, 2004; Wegscheid-Gerlach et al., 2010). One interesting example is that of the ankyrin peptide (AnkP), a potent inhibitor of the major cysteine protease of *P. falciparum*, falcipain-2, which was delivered into parasite-infected red blood cells (*Pf*RBC) via the *Antennapedia* homeoprotein internalization domain; this served to demonstrate not only the antimalarial properties of peptidase substrate analogs like AnkP, but also the potentially useful role of another class of MAP, the cell-penetrating peptides/proteins (CPP), for intracellular delivery of antimalarials (Dhawan et al., 2003). There have been also many reports on synthetic peptides or protein fragments with specific targets at parasite/host or parasite/vector interfaces, for instance, peptide fragments of adhesins that are involved in host-cell invasion (Malpede and Tolia, 2014).

Many broad-spectrum AMP from various sources, including anopheline mosquitoes (malaria vectors), have also been found to exhibit different degrees of antimalarial action (Bell, 2011). The fact that *Plasmodia* are eukaryotes may appear incompatible with the general notion that selective AMP preferentially target negatively-charged prokaryote membranes; however, the phospholipid composition of membranes from intraerythrocytic parasites are markedly different from those of their host eukaryote cells, RBC; moreover, upon infection by *P. falciparum*, RBC undergo significant changes in their membranes, whose composition gets closer to that of parasitic membranes (Hsiao et al., 1991). For instance, as compared to healthy RBC, *Pf*RBC have increased contents of phosphatidylinositol and phosphatidic acid, and decreased contents of sphingomyelin, whereas phosphatidylethanolamines remain more or less unchanged (Hsiao et al., 1991). In other words, *Pf*RBC membranes differ from those of healthy RBC; this, together with the fact that mechanisms of antimalarial action by AMP remain unknown, explains why the paradox of AMP exhibiting antiprotozoal action is only apparent. This comes in agreement with findings from Gelhaus et al. (2008), who found that NK-2, a small cationic AMP, while hardly affecting healthy RBC, promptly internalized *Pf*RBC, affecting the viability of intracellular parasites; studies with liposomes, by the same authors, revealed a phosphatidylserine-dependent lysis by NK-2, which indicates that small cationic AMP may be selective to *Pf*RBC, hence, emerging as a potential new class of antimalarials. In fact, a range of natural and synthetic AMP were found to have promising anti-plasmodial properties (**Table 1**), showing that modulation of the innate immune response is an effective approach to novel peptide anti-infective agents (Haney and Hancock, 2013). Another relevant example is that of phylloseptin-1(PS-1), an AMP capable to control the growth and cause *in vitro* destruction of *P. falciparum*, at the concentration of 16 μg/mL, well-below the levels at which this peptide is toxic to mammalian cells (Kückelhaus et al., 2009). More examples of antimalarial AMP are next revised in higher detail.

**Table 1:** Antimicrobial peptides reported as active against *Plasmodium spp*. parasites.

Peptide	Sequence/origin/reference	Activity
CA(1-13)M(1-13)	KWKLFKKIEKVGQGIGAVLKVLTTGL Cecropin A/melittin hybrid (Frederik et al., 1989)	IC_50_ 10 mM (*Pf*RBC)
Cecropin B	KWKVFKKIEKMGRNIRNGIVKAGPAIAVLGEAKALG *Hyalophora cecropia* (Gwadz et al., 1989)	81–94% abortion of oocyst development (*Plasmodium spp.*) at 0.5 μg/μL (128 μM)
Defensin A	ATCDLLSGFGVGDSACAAHCIARGNRGGYCNSKKVCVCRN *Aedes aegypti* (Kokoza et al., 2010)	∼85% inhibition of oocyst proliferation in transgenic mosquitoes (*P. gallinaceum*)
Dermaseptin DS_3_	*X-*ALWKNMLKGIGKLAGKAALGAVKKLVGAES *Phyllomedusa sauvagii* (dermaseptin derivative) (Ghosh et al., 1997)	IC_50_ 0.8–1.5 μM (*Pf*RBC)
Dermaseptin DS_4_	*X-*ALWMTLLKKVLKAAAKAALNAVLVGANA *Phyllomedusa sauvagii* (dermaseptin derivative) (Ghosh et al., 1997)	IC_50_ 0.27–2.2 μM (*Pf*RBC)
D -HALO-rev	AKKLOHALHOALLALOHLAHOLLAKK *Synthetic* (Mason et al., 2009)	IC_50_ 0.1 μM (*Pf*RBC)
Drosomycin	DCLSGRYKGPCAVWDNETCRRVCKEEGRSSGHCSPSLKC-WCEGC *Drosophila melanogaster* ( Tian et al., 2008)	70% gametocytes inhibition at 20 μM (*P. berghei*)
Gambicin	MVFAYAPTXARXKSIGARYXGYGYLNRKGVSXDGQTTIN-SXEDXKRKFGRXSDGFIT *Anopheles gambiae* ( Vizioli et al., 2001)	54.6% ookinetes killed at 10 μM (*P. berghei*)
IDR-1018	VRLIVAVRIWRR Bactenicin derivative (bovine neutrophils) ( Wieczorek et al., 2010)	Protection against cerebral malaria
Dermaseptin K4K20-S4	ALWKTLLKKVLKAAAKAALKAVLVGANA *Phyllomedusa sauvagii* (dermasepin S4 derivative) (Krugliak et al., 2000)	IC_50_ 0.2 μM (*Pf*RBC)
Dermaseptin K4-S4(1-13)a	ALWMTLLKKVLKA *Phyllomedusa sauvagii* (Dermasepin S4 derivative) (Krugliak et al., 2000)	IC_50_ 6 mM (*Pf*RBC)
Dermaseptin NC7-P	H_2_N-(CH_2_)_6_-CO-ALWKTLLKKVLKA-NH_2_ *Phyllomedusa sauvagii* (dermaseptin K4-S4(1-13)a derivative) (Efron et al., 2002)	IC_50_ 5.3 μM (*Pf*RBC, ring stage); 6.2 μM (*Pf*RBC, trophozoites)
Magainin 2	GIGKFLHSAKKFGKAFVGEIMNS *Xenopus laevis* (Frederik et al., 1989)	82–95% abortion ofoocyst development (*Plasmodium spp.*) at 0.5 μg/mL (203 μM)
NK-2	KILRGVCKKIMRTFLRRISKDILTGKK Synthetic (Gelhaus et al., 2008)	IC_50_ 1–10 μM (*Pf*RBC)
SB-37	MPKWKVFKKIEKVGRNIRNGIVKAGPAIAVLGEAKALG Synthetic cecropin B derivative (Jaynes et al., 1988)	IC_50_ ∼50 μM (*Pf*RBC)
Scorpine	GWINEEKIQKKIDERMGNTVLGGMAKAIVHKMAKNEFQ-CMANMDMLGNCEKHCQTSGEKGYCHGTKCKCGTPLSY *Pandinus imperator* (Conde et al., 2000; Carballar-Lejarazu et al., 2008)	IC_50_: 1 μM (*P. berghei* ookinetes); ∼10 μM (*P. berghei* gametes)
Shiva-1	MPRWRLFRRIDRVGKQIKQGILRAGPAIALVGDARAVG Synthetic cecropin B derivative (Jaynes et al., 1988)	IC_50_ ∼20 μM (*Pf*RBC)
Vida1 Vida 2 Vida 3	KWKKFKKGIGKLFV KWPKFKKGIPWLFV KWPKFRRGIPFLFV Synthetic cecropin B/melittin hybrids (Arrighi et al., 2002)	≥60% mortality of young *P. berghei* ookinetes at 50 μM

#### Cecropins and derivatives

Cecropins, from the moth *Hyalophora cecropia*, disturb the development of oocysts into sporozoites, with a 50% lethal dose between 0.5 and 1 μg/μL (Gwadz et al., 1989). Synthetic derivatives of cecropins have also been produced and analyzed for their effects against malaria: SB-37, highly similar to cecropin B, and Shiva-1, with 40% homology to the same cecropin from *H. cecropia*, were significantly lytic to *P. falciparum* blood stage forms at 50 μM; still, while SB-37 was equipotent to cecropin B, Shiva-1 was twice as active as this cecropin (Jaynes et al., 1988). The effect of another cecropin-like peptide, Shiva-3, on *in vitro* ookinete development and on the early sporogonic stages of *P. berghei* in the midgut of *Anopheles albimanus* mosquitoes was investigated; peptide concentrations of 75 and 100 μM were effective in reducing ookinete production and the number of infected mosquitoes in almost all experiments (Rodriguez et al., 1995).

Hybrids of non-hemolytic cecropins with the potent bee venom toxin melittin have been found to inhibit RBC re-invasion by *P. falciparum*; an example of one such hybrid is that of CA(1-13)M(1-13), which is an order of magnitude more potent than magainin, cecropin A, and cecropin B, and is active in the 5–10 μM range (Frederik et al., 1989). More recent cecropin-melittin hybrids Vida1 (mainly consisting of α-helices), Vida2 (essentially composed by β-sheets), and Vida3 (a combination of coils and sheets) were developed and tested against *P. berghei* and *P. yoelii* gametocytes. The mortality rate of: Vida1 was 65% on young ookinetes (10 h), Vida2 was 60–70% on maturing ookinetes (14 and 24 h), and Vida3 was higher than 60% throughout the entire developmental period (Arrighi et al., 2002). The antimalarial activity of Vida3 was associated to blockage of *Plasmodium* oocyst development, and the peptide was further found as toxic to *Anopheles gambiae* cells at 25 μM (Carter et al., 2013).

The exact molecular mechanisms of membrane activity of cecropins have been under debate for more than 30 years, with two general models being proposed: formation of transmembrane pores, and the carpet model (Melo et al., 2009). A recent study demonstrates that cecropins A and B produce well-defined ion channels of different conductance levels in bilayer lipid membranes; further increase in peptide concentration causes destabilization and subsequent breakdown of the bilayer, showing that formation of pores is a first stage of membrane destabilization by these two cecropins, while accumulation of a dense peptide carpet precedes complete bilayer disintegration (Efimova et al., 2014).

#### Amphibian antimalarial peptides: dermaseptins, magainins

Dermaseptins, from the skin of *Phyllomedusa* frogs, are highly active against intraerythrocytic forms of different *P. falciparum* strains, with IC_50_ values between 0.8 and 2.2 μM (Matsuda and Koyasu, 2000). A truncated dermaseptin derivative was found to exert anti-*P. falciparum* activity within less than 1 min after exposure, involving permeabilization of the host cell membrane (Bell et al., 2006). In order to decrease hemolytic activity of dermaseptins, aminoheptanoyl derivatives were synthesized; a screening against *P. falciparum* revealed higher activity for the more hydrophobic dermaseptin derivatives, some of which showing significant selectivity between antiplasmodial activity versus hemolytic activity (Efron et al., 2002; Gavigan et al., 2003).

Following discovery of antiplasmodial activity of the 28-residue AMP dermaseptin S4 (Ghosh et al., 1997), a derived 13-residue AMP, K4-S4(1-13), was found to display considerable *in vitro* efficacy on *P. falciparum* (Krugliak et al., 2000). The antiplasmodial action of K4-S4(1-13) was fast and shown to be mediated by permeabilization of host cell plasma membrane. Although K4-S4(1-13) was less hemolytic to healthy RBC than to *Pf*RBC, selectivity was not high enough and turned evident the necessity to develop additional derivatives active on parasites but with minimal threat to normal erythrocytes. Recently, acyl derivatives of K4-S4(1-13) were shown to have increased antiplasmodial activity, but the most potent of them was still significantly hemolytic (Dagan et al., 2002).

#### Antimalarial peptides from insect vectors of parasitic diseases

Antimicrobial peptides of insect origin have been found which display antimalarial properties. Drosomycins, isolated from *Drosophila melanogaster*, are an example of insect antimalarial peptides. Drosomycins were tested against development of *P. berghei* ANKA gametocytes, with drosomycin-2 showing 30% inhibition at 20 μM, whereas prototype-peptide drosomycin showed over 70% inhibition at that same concentration (Tian et al., 2008).

The most interesting source of antimalarial AMP concerns insects that are themselves the vectors of parasitic diseases, like anopheline mosquitoes, responsible for malaria transmission. It seems logical that such insects need to be equipped with a considerable defense system against the parasites they carry. In *Anopheles* mosquitoes, there are different known protection mechanisms, such as (i) upregulation of NO synthase, (ii) melanotic encapsulation in refractory mosquitoes that inhibit parasite development (Collins et al., 1986; Vijay et al., 2011), and production of AMP that might play an important role in refractoriness. In the major vector of *P. falciparum* in sub-Saharan Africa, *A. gambiae*, defensin, cecropin, and gambicin AMP have been found (Vizioli et al., 2000; Blandin et al., 2002; Kim et al., 2004).

The *A. gambiae* cecropin gene is mainly expressed in the mosquito midgut in hemocyte-like cells, and its levels are significantly raised within 2 h of infection (Vizioli et al., 2000). The activity of the *A. gambiae* cecropin against *Plasmodium* was studied by creating transgenic mosquitoes with cecA expression under the control of the *Aedes aegypti* carboxypeptidase promotor. The number of oocysts was reduced by 60% compared to the non-transgenic mosquitoes (Kim et al., 2004).

Gambicin, extracted from two *A. gambiae* cell lines, is an immune-induced peptide predominantly expressed in the anterior midgut compartment, thorax, and abdomen. The mature gambicin peptide is active against Gram-positive and Gram-negative bacteria, filamentous fungi, and *P. berghei* ookinetes (Vizioli et al., 2001).

Other mosquito vectors, such as *A. aegypti*, responsible for transmission of dengue and yellow fever viruses, produce AMP with antimalarial activity. *A. aegypti* releases three 40-AA long defensins (Def A to C) and cecropin A in response to bacterial infections (Lowenberger et al., 1995, 1999). In transgenic *A. aegypti* mosquitoes with co-overexpression of *A. aegypti* cecropin A and defensin A, *P. gallinaceum* oocyst proliferation was significantly inhibited as compared to wildtype mosquitoes (Lowenberger, 2001; Kokoza et al., 2010).

The strategy to produce transgenic mosquitoes that heterologously express AMP in order to interrupt *Plasmodium* transmission was also tested for scorpine, an AMP from the venom of *Pandinus imperator* scorpions. Scorpine belongs to a group of ion channel blockers with high activity against *P. berghei* ANKA gametes and ookinetes (ED_50_ of 10 and 0.7 μM, respectively; Conde et al., 2000) that was shown to disrupt the sporogonic development of *P. berghei*. The scorpine gene was introduced into a vector for generation of transgenic flies resistant to infection by *Plasmodia*. The final aim of this work was to incorporate this gene under the promoter of proteolytic enzymes of the mosquito digestive tract, for synthesis and release of toxic peptide(s) into the stomach of freshly fed mosquitoes potentially carrying *Plasmodium* gametes; the presence of recombinant scorpine could be confirmed in transgenic *A. gambiae* cell supernatants (Possani et al., 2002). At low concentrations, recombinant scorpine reduced the number of ookinetes formed after mosquito feeding on *P. berghei*-infected mouse blood. Scorpine had highest effects (98% inhibition) when added during gamete formation and fertilization (Carballar-Lejarazu et al., 2008). In view of this, strategies to deliver AMP into the mosquitoes to interrupt the sporogonic cycle are relevant; one such strategy has been reported that uses symbiotic bacteria that live in the mosquito’s midgut: the transgenetic symbiont *Pantoea agglomerans*, expressing recombinant AMP Shiva-1 and scorpine, completely inhibited *P. falciparum* sporogonic cycle (Wang et al., 2012).

#### Circular AMP with antimalarial properties

**FIGURE 2:**
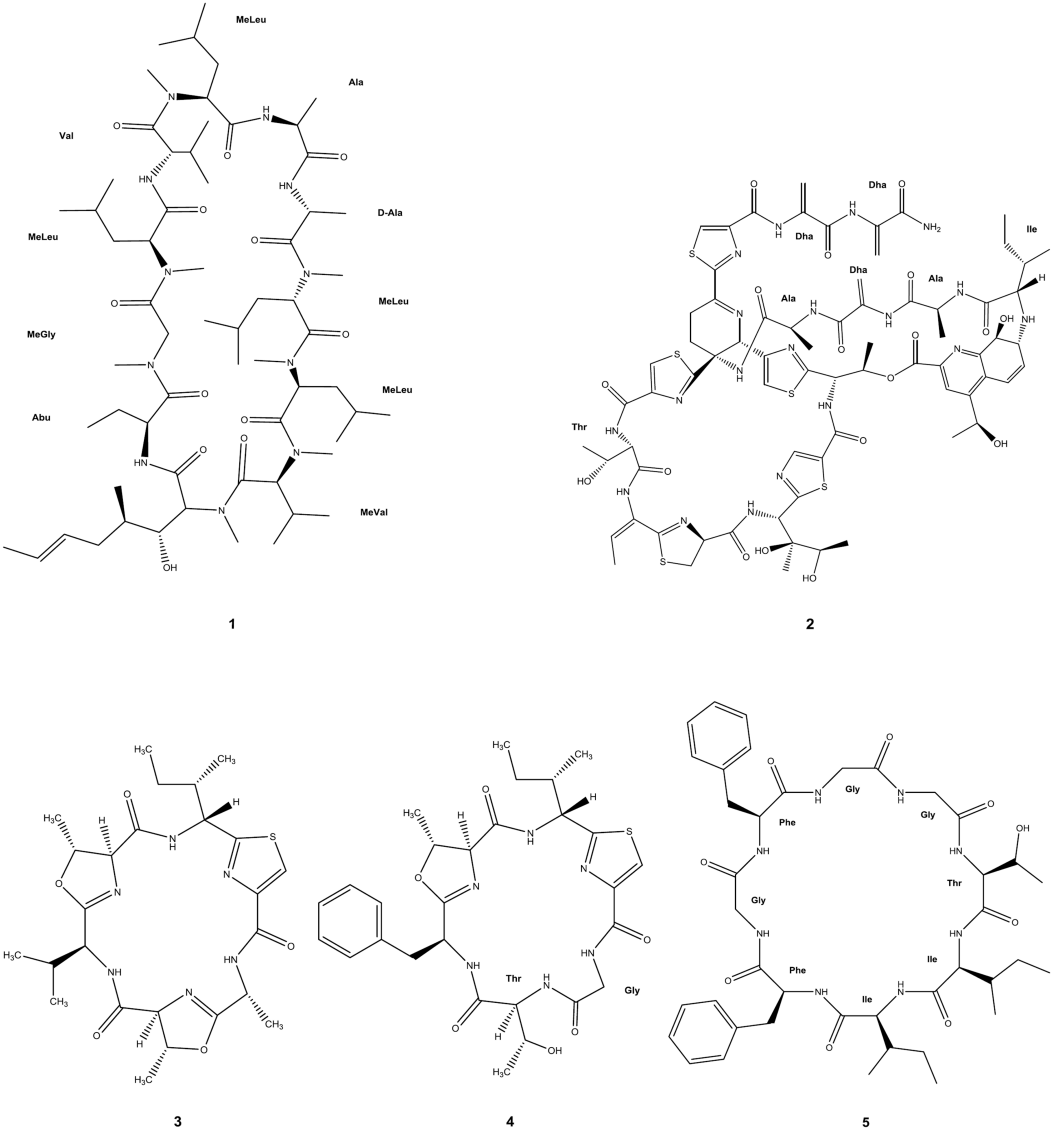
**Circular peptides with antimalarial activity.** Full molecular structure is shown. Amino acid labeling, with the corresponding three-letter code, has been added where applicable: **Abu**, 2-aminobutyric acid; **Ala**, L-alanine; **D-Ala**, D-alanine; **Dha**, dehydroalanine; **Gly**, glycine; **Ile**, L-isoleucine; **Phe**, L-phenylalanine; **Thr**, L-threonine; **Val**, L-valine; **MeGly**, *N*-methylglycine [also known as sarcosine (Sar)]; **MeLeu**, *N*-methylleucine; **MeVal**, *N*-methylvaline. Uncommon amino acid residues or their analogs not possessing any internationally approved abbreviation were kept unlabeled.

There is a significant number of cyclic AMP and derived macrocycles that have shown antimalarial properties. The potentially large, but structurally often well-defined conformational space sampled, combined with the variety of AA, renders cyclic peptides ideally suited to interact with many receptors or to interfere with protein/protein interactions (Katoh et al., 2011; Giordanetto and Kihlberg, 2014). In this context, cyclosporin A (CsA, 1 in **Figure 2**) is a well-characterized immunosuppressant hydrophobic peptide (Borel et al., 1996) that was earlier found to have antimalarial activity against *P. berghei* and *P. yoelii* rodent malaria and on cultured *P. berghei, P. falciparum,* and *P. vivax* parasites (Nickell et al., 1982). More recently, nine CsA-resistant *P. falciparum* clones were isolated, of which three had lesions in cyclophilin genes and two in calcineurin-subunit genes (Kumar et al., 2005). The two cyclophilins affected had been identified as cyclosporin-binding proteins in *P. falciparum* (Gavigan et al., 2003), but CsA may also have other targets in *Plasmodia*, since, it has a number of different known targets including the mammalian P-glycoprotein transporter. In agreement with this hypothesis, sequence polymorphisms in (and possibly expression levels of) a *P. falciparum* P-glycoprotein homolog were found to affect susceptibility to CsA (Gavigan et al., 2007).

Thiostrepton (2 in **Figure 2**), a cyclic thiopeptide, has reported IC_50_ ranging from 1.8 to 17 μM against parasite growth and protein synthesis of *P. falciparum*, though this is suggested to be an overestimate since solubility of the compound in culture medium is poor (Clough et al., 1997; McConkey et al., 1997). IC_50_ values for thiostrepton derivatives were 0.77 μM and above against *P. falciparum* (Schoof et al., 2010), and other thiopeptides were described as displaying nanomolar IC_50_ against organellar protein synthesis in the same strain: micrococcin (3 nM), GE2270A (300 nM), and amythiamicin A (10 nM; Clough et al., 1999).

Most recently, three new macro-heterocyclic AMP, balgacyclamides A–C, were isolated from *Microcystis aeruginosa* EAWAG 251 and thoroughly characterized. Balgacyclamides A (3 in **Figure 2**) and B (4 in **Figure 2**) were evaluated for their antiparasitic activity and found to display micromolar IC_50_ activity against the chloroquine-resistant strain K1 of *P. falciparum* (9.0 and 8.2 μM, respectively) with good selectivity compared to their cytotoxicity (Portmann et al., 2014).

Another recent report on a cyclic antimalarial peptide concerns the Gly-rich cyclic octapeptide pohlianin C (5 in **Figure 2**), whose synthesis provided confirmation of the structure of this natural product. Evaluation against *P. falciparum* showed moderate antiplasmodial activity, consistent with data obtained from the natural sample. In addition, the synthesis of three analogs revealed that the antiplasmodial activity of pohlianin C can be preserved or increased with simplified structures (Lawer et al., 2014).

#### Other synthetic antimalarial AMP: from NK-2 to IDR-1018

NK-2 was one of the first synthetic AMP found to have anti-parasitic properties. NK-2 is a shortened version of the mammalian protein NK-lysin, comprising its residues 39–65, and long known to display lytic activity against the fungal pathogen *Candida albicans* and a variety of Gram-positive and Gram-negative bacteria, while exhibits virtually no hemolytic or cytotoxic activity against human cells (Andrä and Leippe, 1999). In a study aimed at determining the antimicrobial spectrum of both NK-lysin and NK-2, activity against the protozoan parasite *Trypanosoma cruzi* was observed (Jacobs et al., 2003). Later, NK-2 was found to be hemolytic to *Pf*RBC above ∼1 μM, while harmless for healthy RBC up to 10 μM, along with evidence that parasite membrane also suffered permeabilization at 5 and 10 μM. The selective lytic activity of NK-2 on *Pf*RBC was further demonstrated by observation that fluorescently labeled NK-2 binds to infected erythrocytes and to parasites, but not to healthy erythrocytes (Gelhaus et al., 2008).

Another synthetic AMP with antimalarial activity is D-HALO-rev. This design peptide possesses 26 AA with an even distribution of hydrophobic and charged residues, including non-proteinogenic ornithine (O, Orn), a Lys homolog. D-HALO-rev shows an IC_50_ value of 0.1 μM against erythrocytic stages of *P. falciparum*, being able to penetrate *Pf*RBC at sublytic concentrations and kill intraerythrocytic parasites (Mason et al., 2009). Similar results were obtained for an analog resulting from incorporation of Pro, Phe, and D-AA residues (D-Halo-P8F-rev), which showed reduced toxicity toward healthy RBC and fibroblasts (Mason et al., 2009).

A further step forward in this field was recent disclosure of the ability of synthetic peptide IDR-1018 to provide protection against cerebral malaria (Achtman et al., 2012). IDR-1018 is a 12-residue innate defense regulator analog of bactenecin, a cathelicidine from bovine neutrophils. It putatively owes its action to translocation across cell membrane and impairment of an intracellular target (Wieczorek et al., 2010). IDR-1018 was selected for screening in cerebral malaria given its anti-inflammatory capabilities and low toxicity, and found to protect 56% of infected mice from cerebral malaria after prophylactic intravenous administration. When combined with the antimalarial pyrimethamine-chloroquine medicine, IDR-1018 boosted protection from cerebral malaria in 41–68% of infected mice (Achtman et al., 2012).

### PROMISES AND PITFALLS OF PEPTIDE ANTIMALARIALS: FROM CONVENTIONAL PHARMACEUTICAL TO BIOTECHNOLOGICAL APPROACHES

Examples of peptide drug candidates entering clinical trials remain scarce, as compared to those of small molecular drug candidates; despite a few such peptides display antimalarial activity (pexiganan in phase III, omiganan and OP-145 in phase 2, and NVB302 in phase I), their involvement in clinical trials is directed to disorders other than malaria (Fox, 2013). This is mainly due to the traditionally cautious attitude of pharma companies toward peptide-based drugs, given that:

• peptides seldom are orally bioavailable given their usually high molecular weight, extensive enzymatic degradation (proteolysis) and binding to plasma proteins;
• peptide production costs are high and synthesis scale-up is not easy, which will result in medicines too expensive, especially if targeted at low-income countries.

Therefore, until recent years, pharma companies have mostly put their bets on small drug candidates, with molecular weights tipically below 500 Da and oral bioavailability, leaving much larger biomolecule-based candidates put aside, as molecules with over 5000 Da are not orally bioavailable, among other limitations. However, small drugs often suffer from reduced selectivity, leading to unwanted off-target side effects; in turn, peptides and proteins are usually characterized by an extraordinary specificity for their targets, which may largely compensate for their low bioavailability, poor permeability and susceptibility to metabolic inactivation (Craik et al., 2013).

In the particular case of malaria, from World War II to the present, a huge plethora of small molecules nicely obeying the Lipinski’s rule of 5 for orally bioavailable drugs (Lipinski et al., 1997) has been continuously feeding the antimalarial drug pipeline (Teixeira et al., 2014). Nonetheless, control of the disease remains out of reach, especially due to toxicity and resistance issues (Wells and Poll, 2010). Moreover, Lipinski’s rule applies only to drug absorption by passive diffusion through cell membranes, i.e., it is not predictive for compounds actively transported by transmembranar proteins (Leeson, 2012). Altogether, this emphasizes the need for highly specific antimalarials, preferably with no or reduced propensity to elicit parasite resistance, and also that unconventional drugs, like peptides, may eventually fill such need, provided suitable strategies to overcome some challenges posed by peptide drugs are found. Fortunately, the 21st century emerged along with the first evidences of a paradigm shift in this field: between 2011 and 2012, nineteen peptide drugs were approved in the US, and the remarkable expansion of peptide therapeutics development in the late 1990s and 2000s led to an unprecedented number of marketing approvals in 2012, while providing a robust pipeline that should deliver numerous approvals during the remainder of the 2010s; in the US, annual sales of peptide drugs exceed 13 bilion dollars, representing 1.5% of global drug sales. In Europe, Germany and the UK account for 63% of the peptide therapeutics market, with France, Italy, Scandinavia, and Spain making up the rest of the major European salers in this area (Kaspar and Reichert, 2013).

In addition to the above, peptide and protein biotechnology continues evolving at full speed, with the most prominent example today being that of therapeutic antibodies (Leavy, 2010). Other biotechnological approaches include, e.g., bioactive peptide grafting onto suitable biocompatible carriers to enhance peptide bioavailability at the site of action (Costa et al., 2011; Lax and Meenan, 2012a,b; Maia et al., 2014), or development of long-acting release forms of peptides such as somatostatin analogs through encapsulation in biodegradable polymers requiring injection only at extended intervals to raise patient’s compliance (Anthony and Freda, 2009). Genetic engineering and recombinant biotechnology are changing pharma’s perspective toward peptide therapeutics, as genetically engineered proteins offered a window to previously untreatable medical conditions that convinced this industry to conform with the need to develop drugs that could not be administered through the oral route; it also motivated an intense search for alternative (to injection) drug delivery platforms to meet patient’s acceptability, and offers an useful alternative to chemical production of large (>40 AA) peptides, whose synthesis is challenging and expensive, even at low scale; hence, recombinant biotechnology will play an increasingly important role in peptide manufacturing mainly due to quantity requirements (Lax, 2010). Still, biotechnology will complement, and not replace, chemical peptide synthesis in the production of peptide therapeutics: cost of large-scale peptide chemical synthesis, in particular through the most popular solid-phase procedures based on Fmoc-chemistry, has significantly decreased over the past 15 years, mainly due to technological evolution of automated synthesizers and chromatographic systems; moreover, many highly specific and potent peptide therapeutics require daily doses of only a few micrograms; furthermore, when applicable, chemical peptide synthesis is more easily scalable for manufacturing at up to a multi-10 kg or 100-kg scale, and is less demanding in terms of process development, and less personnel intensive in terms of production, quality assurance and regulatory affairs; last, but not least, chemical approaches are far superior to biotechnological ones regarding flexibility in the design of analogs requiring unnatural amino acids or non-proteogenic building blocks (Lax, 2010).

In summary, peptide therapeutics are reaching a maturity level that emphasizes their potential interest against a wide diversity of medical challenges. While, about half of the peptides in clinical trials target indications in oncology, metabolic, cardiovascular and infectious diseases, the total range of therapeutic areas addressed encompasses a wide assortment of medical disorders from endocrinological lesions through to pain and hematology (Kaspar and Reichert, 2013). In the particular case of malaria, development of peptide therapeutics is still at its earliest infancy, but several cutting-edge approaches to antimalarial peptides are emerging today. One recent example followed demonstration that *Pf*SERA5 plays an important role in parasite development; in this study, a number of peptides from the *N*- and *C*-terminal regions of *Pf*SERA5 active domain was synthesized and evaluated as antiplasmodials. These peptides reduced activity of the recombinant enzyme and co-localized with *Pf*SERA5 within the parasite, thereby indicating the specific inhibition of *Pf*SERA5 activity. Such results reinforce the role of *Pf*SERA5 for the intraerythrocytic development of malaria parasites and unveil the relevance of this enzyme as target for new peptide antimalarials (Kanodia et al., 2014). In view of this, and considering the recent unveiling of the *P. falciparum* genome (Boddey et al., 2013), we can only expect the future to confirm that a new class of antimalarial drugs, based on AMP, will rise.

## CONCLUDING REMARKS

This review highlights that peptides are being put forward as one potential novel class of antimalarial drugs. A range of AMP exhibit antimalarial activity on malarial parasites in their blood or mosquito stages or both. Some peptides naturally occurring in mosquitoes affect the parasites transmitted by those insect vectors, but it has been difficult to determine the magnitude of their effects in the natural setting. However, this field of research is a recent one, and more systematic studies are needed to identify the structural variants that are most potent and selective on cultured parasites and to test them *in vivo*. Better understanding of mechanisms of action would help to guide the design of new peptides and the development of *in vitro* assays with which to compare their target-binding affinities. Some antimalarial peptides are believed to act selectively on infected erythrocyte and/or intraerythrocytic parasite membranes, in which case the appropriate model membrane systems are required. This means that another well-known family of MAP, besides AMP, may soon gain also relevance in antimalarial approaches: that of CPP. These can translocate into cells without causing membrane damage, therefore being useful carriers for therapeutic cargoes to treat various conditions (De Figueiredo et al., 2014). Membrane-active AMP and CPP show significant similarities in charge, structure, and initial steps of interactions with membranes; moreover, CPP are being identified with antimalarial activity *per se*, e.g., TP10 that has broad-spectrum activity against both blood and mosquito stages of *P. falciparum* (Arrighi et al., 2008). In conclusion, MAP seem to be paving the way toward establishment of useful peptide-based drugs against parasitic infections.

## Conflict of Interest Statement

The authors declare that the research was conducted in the absence of any commercial or financial relationships that could be construed as a potential conflict of interest.

## References

[B1] Achtman A. H. Pilat S. Law C. W. Lynn D. J. Janot L. Mayer M. L. (2012). Effective adjunctive therapy by an innate defense regulatory peptide in a preclinical model of severe malaria. *Sci. Transl. Med.* 4 135ra164. 10.1126/scitranslmed.3003515 22623740

[B2] Agerberth B. Charo J. Werr J. Olsson B. Idali F. Lindbom L. (2000). The human antimicrobial and chemotactic peptides ll-37 and alpha-defensins are expressed by specific lymphocyte and monocyte populations. *Blood* 96 3086–3093. 11049988

[B3] Andrä J. Leippe M. (1999). Candidacidal activity of shortened synthetic analogs of amoebapores and NK-lysin. *Med. Microbiol. Immunol.* 188 117–124 10.1007/s004300050113 10776841

[B4] Anthony L. Freda P. U. (2009). From somatostatin to octreotide LAR: evolution of a somatostatin analogue. *Curr. Med. Res. Opin.* 25 2989–2999 10.1185/03007990903328959 19842996 PMC3678951

[B5] Arrighi R. B. Ebikeme C. Jiang Y. Ranford-Cartwright L. Barrett M. P. Langel U. (2008). Cell-penetrating peptide TP10 shows broad-spectrum activity against both *Plasmodium falciparum* and *Trypanosoma brucei brucei*. *Antimicrob. Agents Chemother.* 52 3414–3417 10.1128/AAC.01450-07 18519720 PMC2533502

[B6] Arrighi R. B. Nakamura C. Miyake J. Hurd H. Burgess J. G. (2002). Design and activity of antimicrobial peptides against sporogonic-stage parasites causing murine malarias. *Antimicrob. Agents Chemother.* 46 2104–2110 10.1128/AAC.46.7.2104-2110.2002 12069961 PMC127320

[B7] Bals R. Wang X. Zasloff M. Wilson J. M. (1998). The peptide antibiotic ll-37/hcap-18 is expressed in epithelia of the human lung where it has broad antimicrobial activity at the airway surface. *Proc. Natl. Acad. Sci. U.S.A.* 95 9541–9546 10.1073/pnas.95.16.9541 9689116 PMC21374

[B8] Bastos M. Bai G. Gomes P. Andreu D. Goormaghtigh E. Prieto M. (2008). Energetics and partition of two cecropin-melittin hybrid peptides to model membranes of different composition. *Biophys. J.* 94 2128–2141 10.1529/biophysj.107.119032 18032555 PMC2257877

[B9] Bechinger B. (1996). Towards membrane protein design: pH dependent topology of histidine-containing polypeptides. *J. Mol. Biol.* 263 768–775 10.1006/jmbi.1996.0614 8947574

[B10] Becker K. Tilley L. Vennerstrom J. L. Roberts D. Rogerson S. Ginsburg H. (2004). Oxidative stress in malaria parasite-infected erythrocytes: host-parasite interactions. *Int. J. Parasitol.* 34 163–189 10.1016/j.ijpara.2003.09.011 15037104

[B11] Beevers A. J. Dixon A. M. (2010). Helical membrane peptides to modulate cell function. *Chem. Soc. Rev.* 39 2146–2157 10.1039/b912944h 20502803

[B12] Bell A. (2011). Antimalarial peptides: the long and the short of it. *Curr. Pharm. Des.* 17 2719–2731 10.2174/138161211797416057 21728986

[B13] Bell A. Monaghan P. Page A. P. (2006). Peptidyl-prolyl cis-trans isomerases (immunophilins) and their roles in parasite biochemistry, host-parasite interaction and antiparasitic drug action. *Int. J. Parasitol.* 36 261–276 10.1016/j.ijpara.2005.11.003 16443228

[B14] Bellamy W. Takase M. Yamauchi K. Wakabayashi H. Kawase K. Tomita M. (1992). Identification of the bactericidal domain of lactoferrin. *Biochim. Biophys. Acta* 1121 130–136 10.1016/0167-4838(92)90346-F 1599934

[B15] Biagini G. A. O’Neill P. M. Nzila A. Ward S. A. Bray P. G. (2003). Antimalarial chemotherapy: young guns or back to the future? *Trends Parasitol.* 19 479–487 10.1016/j.pt.2003.09.011 14580958

[B16] Blackman M. J. (2004). Proteases in host cell invasion by the malaria parasite. *Cell. Microbiol.* 6 893–903 10.1111/j.1462-5822.2004.00437.x 15339265

[B17] Blandin S. Moita L. F. Kocher T. Wilm M. Kafatos F. C. Levashina E. A. (2002). Reverse genetics in the mosquito *Anopheles gambiae*: targeted disruption of the defensin gene. *EMBO Rep.* 3 852–856 10.1093/embo-reports/kvf180 12189180 PMC1084233

[B18] Boddey J. A. Carvalho T. G. Hodder A. N. Sargeant T. J. Sleebs B. E. Marapana D. (2013). Role of Plasmepsin V in export of diverse protein families from the *Plasmodium falciparum* exportome. *Traffic* 14 532–550 10.1111/tra.12053 23387285

[B19] Boman H. G. (2003). Antibacterial peptides: basic facts and emerging concepts. *J. Intern. Med.* 254 197–215 10.1046/j.1365-2796.2003.01228.x 12930229

[B20] Boman H. G. Wade D. Boman I. A. Wahlin B. Merrifield R. B. (1989). Antibacterial and antimalarial properties of peptides that are cecropin-melittin hybrids. *FEBS Lett.* 259 103–106 10.1016/0014-5793(89)81505-4 2689223

[B21] Borel J. F. Baumann G. Chapman I. Donatsch P. Fahr A. Mueller E. A. (1996). In vivo pharmacological effects of ciclosporin and some analogues. *Adv. Pharmacol.* 35 115–246 10.1016/S1054-3589(08)60276-8 8920206

[B22] Bruston F. Lacombe C. Zimmermann K. Piesse C. Nicolas P. El Amri C. (2007). Structural malleability of plasticins: preorganized conformations in solution and relevance for antimicrobial activity. *Biopolymers* 86 42–56 10.1002/bip.20703 17309077

[B23] Bulet P. Hetru C. Dimarcq J. L. Hoffmann D. (1999). Antimicrobial peptides in insects; structure and function. *Dev. Comp. Immunol.* 23 329–344 10.1016/S0145-305X(99)00015-4 10426426

[B24] Carballar-Lejarazu R. Rodriguez M. H. de la Cruz Hernandez–Hernandez F. Ramos-Castaneda J. Possani L. D. Zurita-Ortega M. (2008). Recombinant scorpine: a multifunctional antimicrobial peptide with activity against different pathogens. *Cell. Mol. Life Sci.* 65 3081–3092 10.1007/s00018-008-8250-8 18726072 PMC11131772

[B25] Carlsson A. Engstrom P. Palva E. T. Bennich H. (1991). Attacin, an antibacterial protein from *Hyalophora cecropia*, inhibits synthesis of outer membrane proteins in *Escherichia coli* by interfering with omp gene transcription. *Infect. Immun.* 59 3040–3045. 1715318 10.1128/iai.59.9.3040-3045.1991 PMC258132

[B26] Carlsson A. Nystrom T. de Cock H. Bennich H. (1998). Attacin – an insect immune protein-binds lps and triggers the specific inhibition of bacterial outer-membrane protein synthesis. *Microbiology* 144 2179–2188 10.1099/00221287-144-8-2179 9720039

[B27] Carter V. Underhill A. Baber I. Sylla L. Baby M. Larget-Thiery I. (2013). Killer bee molecules: antimicrobial peptides as effector molecules to target sporogonic stages of *Plasmodium*. *PLoS Pathog.* 9:e1003790 10.1371/journal.ppat.1003790 PMC3836994 24278025

[B28] Castle M. Nazarian A. Yi S. S. Tempst P. (1999). Lethal effects of apidaecin on *Escherichia coli* involve sequential molecular interactions with diverse targets. *J. Biol. Chem.* 274 32555–32564 10.1074/jbc.274.46.32555 10551808

[B29] Christensen B. Fink J. Merrifield R. B. Mauzerall D. (1988). Channel-forming properties of cecropins and related model compounds incorporated into planar lipid membranes. *Proc. Natl. Acad. Sci. U.S.A.* 85 5072–5076 10.1073/pnas.85.14.5072 2455891 PMC281690

[B30] Clough B. Rangachari K. Strath M. Preiser P. R. Wilson R. J. (1999). Antibiotic inhibitors of organellar protein synthesis in *Plasmodium falciparum*. *Protist* 150 189–195 10.1016/S1434-4610(99)70021-0 10505418

[B31] Clough B. Strath M. Preiser P. Denny P. Wilson I. R. (1997). Thiostrepton binds to malarial plastid rRNA. *FEBS Lett.* 406 123–125 10.1016/S0014-5793(97)00241-X 9109400

[B32] Collins F. H. Sakai R. K. Vernick K. D. Paskewitz S. Seeley D. C. Miller L. H. (1986). Genetic selection of a *Plasmodium*-refractory strain of the malaria vector *Anopheles gambiae*. *Science* 234 607–610 10.1126/science.3532325 3532325

[B33] Conde R. Zamudio F. Z. Rodriguez M. H. Possani L. D. (2000). Scorpine, an anti-malaria and anti-bacterial agent purified from scorpion venom. *FEBS Lett.* 471 165–168 10.1016/S0014-5793(00)01384-3 10767415

[B34] Costa F. Carvalho I. F. Montelaro R. C. Gomes P. Martins M. C. (2011). Covalent immobilization of Antimicrobial Peptides (AMPs) onto biomaterial surfaces. *Acta Biomater.* 7 1431–1440 10.1016/j.actbio.2010.11.005 21056701

[B35] Cotter P. D. Ross R. P. Hill C. (2013). Bacteriocins – a viable alternative to antibiotics? *Nat. Rev. Microbiol.* 11 95–105 10.1038/nrmicro2937 23268227

[B36] Craik D. J. Conibear A. C. (2011). The chemistry of cyclotides. *J. Org. Chem.* 76 4805–4817 10.1021/jo200520v 21526790

[B37] Craik D. J. Daly N. L. Bond T. Waine C. (1999). Plant cyclotides: a unique family of cyclic and knotted proteins that defines the cyclic cystine knot structural motif. *J. Mol. Biol.* 294 1327–1336 10.1006/jmbi.1999.3383 10600388

[B38] Craik D. J. Fairlie D. P. Liras S. Price D. (2013). The future of peptide-based drugs. *Chem. Biol. Drug Des.* 81 136–147 10.1111/cbdd.12055 23253135

[B39] Dagan A. Efron L. Gaidukov L. Mor A. Ginsburg H. (2002). In Vitro Antiplasmodium effects of dermaseptin S4 derivatives. *Antimicrob. Agents Chemother.* 46 1059–1066 10.1128/AAC.46.4.1059-1066.2002 11897590 PMC127115

[B40] De Figueiredo I. R. Freire J. M. Flores L. Veiga A. S. Castanho M. A. (2014). Cell-penetrating peptides: a tool for effective delivery in gene-targeted therapies. *IUBMB Life* 66 182–194 10.1002/iub.1257 24659560

[B41] Dhawan S. Dua M. Chishti A. H. Hanspal M. (2003). Ankyrin peptide blocks falcipain-2-mediated malaria parasite release from red blood cells. *J. Biol. Chem.* 278 30180–30186 10.1074/jbc.M305132200 12775709

[B42] Dixon M. W. Thompson J. Gardiner D. L. Trenholme K. R. (2008). Sex in plasmodium: a sign of commitment. *Trends Parasitol.* 24 168–175 10.1016/j.pt.2008.01.004 18342574

[B43] Dondorp A. M. Nosten F. Yi P. Das D. Phyo A. P. Tarning J. (2009). Artemisinin resistance in *Plasmodium falciparum* malaria. *N. Engl. J. Med.* 361 455–467 10.1056/NEJMoa0808859 19641202 PMC3495232

[B44] Eckstein-Ludwig U. Webb R. J. Van Goethem I. D. East J. M. Lee A. G. Kimura M. (2003). Artemisinins target the SERCA of *Plasmodium falciparum*. *Nature* 424 957–961 10.1038/nature01813 12931192

[B45] Efimova S. S. Schagina L. V. Ostroumova O. S. (2014). Channel-forming activity of cecropins in lipid bilayers: effect of agents modifying the membrane dipole potential. *Langmuir* 30 7884–7892 10.1021/la501549v 24969512

[B46] Efron L. Dagan A. Gaidukov L. Ginsburg H. Mor A. (2002). Direct interaction of dermaseptin S4 aminoheptanoyl derivative with intraerythrocytic malaria parasite leading to increased specific antiparasitic activity in culture. *J. Biol. Chem.* 277 24067–24072 10.1074/jbc.M202089200 11937508

[B47] Ekengren S. Hultmark D. (1999). *Drosophila* cecropin as an antifungal agent. *Insect Biochem. Mol. Biol.* 29 965–972 10.1016/S0965-1748(99)00071-5 10560137

[B48] Fjell C. D. Hiss J. A. Hancock R. E. Schneider G. (2011). Designing antimicrobial peptides: form follows function. *Nat. Rev. Drug Discov.* 11 37–51 10.1038/nrd3591 22173434

[B49] Flannery E. L. Chatterjee A. K. Winzeler E. A. (2013). Antimalarial drug discovery – approaches and progress towards new medicines. *Nat. Rev. Microbiol.* 11 849–862 10.1038/nrmicro3138 24217412 PMC3941073

[B50] Fox J. L. (2013). Antimicrobial peptides stage a comeback. *Nat. Biotechnol.* 31 379–382 10.1038/nbt.2572 23657384

[B51] Frederik P. M. Stuart M. C. Bomans P. H. Busing W. M. (1989). Phospholipid, nature’s own slide and cover slip for cryo-electron microscopy. *J. Microsc.* 153 81–92 10.1111/j.1365-2818.1989.tb01469.x 2709403

[B52] Ganz T. Weiss J. (1997). Antimicrobial peptides of phagocytes and epithelia. *Semin. Hematol.* 34 343–354. 9347585

[B53] Gavigan C. S. Kiely S. P. Hirtzlin J. Bell A. (2003). Cyclosporin-binding proteins of *Plasmodium falciparum*. *Int. J. Parasitol.* 33 987–996 10.1016/S0020-7519(03)00125-5 12906882

[B54] Gavigan C. S. Shen M. Machado S. G. Bell A. (2007). Influence of the *Plasmodium falciparum* P-glycoprotein homologue 1 (pfmdr1 gene product) on the antimalarial action of cyclosporin. *J. Antimicrob. Chemother.* 59 197–203 10.1093/jac/dkl461 17105736

[B55] Ge Y. MacDonald D. L. Holroyd K. J. Thornsberry C. Wexler H. Zasloff M. (1999). In vitro antibacterial properties of pexiganan, an analog of magainin. *Antimicrob. Agents Chemother.* 43 782–788. 10103181 10.1128/aac.43.4.782 PMC89207

[B56] Gelhaus C. Jacobs T. Andra J. Leippe M. (2008). The antimicrobial peptide NK-2, the core region of mammalian nk-lysin, kills intraerythrocytic *Plasmodium falciparum*. *Antimicrob. Agents Chemother.* 52 1713–1720 10.1128/AAC.01342-07 18332165 PMC2346629

[B57] Ghosh J. K. Shaool D. Guillaud P. Ciceroni L. Mazieri D. Kustanovich I. (1997). Selective cytotoxicity of dermaseptin S3 toward intraerythrocytic *Plasmodium falciparum* and the underlying molecular basis. *J. Biol. Chem.* 272 31609–31616 10.1074/jbc.272.50.31609 9395500

[B58] Giordanetto F. Kihlberg J. (2014). Macrocyclic drugs and clinical candidates: what can medicinal chemists learn from their properties? *J. Med. Chem.* 57 278–295 10.1021/jm400887j 24044773

[B59] González C. Langdon G. M. Bruix M. Gálvez A. Valdivia E. Maqueda M. (2000). Bacteriocin AS-48, a microbial cyclic polypeptide structurally and functionally related to mammalian NK-lysin. *Proc. Natl. Acad. Sci. U.S.A.* 97 11221–11226 10.1073/pnas.210301097 11005847 PMC17181

[B60] Gwadz R. W. Kaslow D. Lee J. Y. Maloy W. L. Zasloff M. Miller L. H. (1989). Effects of magainins and cecropins on the sporogonic development of malaria parasites in mosquitos. *Infect. Immun.* 57 2628–2633. 2759705 10.1128/iai.57.9.2628-2633.1989 PMC313504

[B61] Haney E. F. Hancock R. E. W. (2013). Peptide design for antimicrobial and immunomodulatory applications. *Biopolymers* 100 572–583 10.1002/bip.22250 23553602 PMC3932157

[B62] Hsiao L. L. Howard R. J. Aikawa M. Taraschi T. F. (1991). Modification of host cell membrane lipid composition by the intra-erythrocytic human malaria parasite *Plasmodium falciparum*. *Biochem. J.* 274 121–132. 2001227 10.1042/bj2740121 PMC1149929

[B63] Hsu C. H. Chen C. Jou M. L. Lee A. Y. Lin Y. C. Yu Y. P. (2005). Structural and DNA-binding studies on the bovine antimicrobial peptide, indolicidin: evidence for multiple conformations involved in binding to membranes and DNA. *Nucleic Acids Res.* 33 4053–4064 10.1093/nar/gki725 16034027 PMC1179735

[B64] Jacobs T. Bruhn H. Gaworski I. Fleischer B. Leippe M. (2003). NK-lysin and its shortened analog NK-2 exhibit potent activities against *Trypanosoma cruzi*. *Antimicrob. Agents Chemother.* 47 607–613 10.1128/AAC.47.2.607-613.2003 12543667 PMC151766

[B65] Jagadish K. Camarero J. A. (2010). Cyclotides: a promising scaffold for peptide-based therapeutics. *Biopolymers* 94 611–616 10.1002/bip.21433 20564025 PMC3000894

[B66] Jaynes J. M. Burton C. A. Barr S. B. Jeffers G. W. Julian G. R. White K. L. (1988). In vitro cytocidal effect of novel lytic peptides on *Plasmodium falciparum* and *Trypanosoma cruzi*. *FASEB J.* 2 2878–2883. 3049204 10.1096/fasebj.2.13.3049204

[B67] Jeu L. Fung H. B. (2004). Daptomycin: a cyclic lipopeptide antimicrobial agent. *Clin. Ther.* 2 1728–1757 10.1016/j.clinthera.2004.11.014 15639687 PMC7133638

[B68] Kanodia S. Kumar G. Rizzi L. Pedretti A. Hodder A. N. Romeo S. (2014). Synthetic peptides derived from the C-terminal 6 kDa region of *Plasmodium falciparum* SERA5 inhibit the enzyme activity and malaria parasite development. *Biochim. Biophys. Acta* 1840 2765–2775 10.1016/j.bbagen.2014.04.013 24769454

[B69] Kaspar A. A. Reichert J. M. (2013). Future directions for peptide therapeutics developments. *Drug Discov. Today* 18 807–817 10.1016/j.drudis.2013.05.011 23726889

[B70] Katoh T. Goto Y. Reza M. S. Suga H. (2011). Ribosomal synthesis of backbone macrocyclic peptides. *Chem. Commun.* 47 9946–9958 10.1039/c1cc12647d 21766105

[B71] Kim W. Koo H. Richman A. M. Seeley D. Vizioli J. Klocko A. D. (2004). Ectopic expression of a cecropin transgene in the human malaria vector mosquito *Anopheles gambiae* (diptera: Culicidae): effects on susceptibility to *Plasmodium*. *J. Med. Entomol.* 41 447–455 10.1603/0022-2585-41.3.447 15185949

[B72] Klein E. Y. (2013). Antimalarial drug resistance: a review of the biology and strategies to delay emergence and spread. *Int. J. Antimicrob. Agents* 41 311–317 10.1016/j.ijantimicag.2012.12.007 23394809 PMC3610176

[B73] Kokoza V. Ahmed A. Shin S. W. Okafor N. Zou Z. Raikhel A. S. (2010). Blocking of *Plasmodium* transmission by cooperative action of cecropin a and defensin a in transgenic *Aedes aegypti* mosquitoes. *Proc. Natl. Acad. Sci. U.S.A.* 107 8111–8116 10.1073/pnas.1003056107 20385844 PMC2889521

[B74] Krugliak M. Feder R. Zolotarev V. Gaidukov L. Dagan A. Ginsburg H. (2000). Antimalarial activities of dermaseptin S4 derivatives. *Antimicrob. Agents Chemother.* 44 2442–2451 10.1128/AAC.44.9.2442-2451.2000 10952593 PMC90083

[B75] Kückelhaus S. A. S. Leite J. R. Muniz-Junqueira M. I. Sampaio R. N. Bloch C. Jr. Tosta C. E. (2009). Antiplasmodial and antileishmanial activities of phylloseptin-1, an antimicrobial peptide from the skin secretion of *Phyllomedusa azurea* (Amphibia). *Exp. Parasitol.* 123 11–16 10.1016/j.exppara.2009.05.002 19460376

[B76] Kumar R. Musiyenko A. Barik S. (2005). *Plasmodium falciparum* calcineurin and its association with heat shock protein 90: mechanisms for the antimalarial activity of cyclosporin A and synergism with geldanamycin. *Mol. Biochem. Parasitol.* 141 29–37 10.1016/j.molbiopara.2005.01.012 15811524

[B77] Kuroda K. Gaputo G. A. (2013). Antimicrobial polymers as synthetic mimics of host-defense peptides. *Wiley Interdiscip. Rev. Nanomed. Nanobitechnol.* 5 49–66 10.1002/wnan.1199 23076870

[B78] Ladokhin A. S. White S. H. (2001). Protein chemistry at membrane interfaces: non-additivity of electrostatic and hydrophobic interactions. *J. Mol. Biol.* 309 543–552 10.1006/jmbi.2001.4684 11397078

[B79] Lawer A. Tai J. Jolliffe K. A. Fletcher S. Avery V. M. Hunter L. (2014). Total synthesis and antiplasmodial activity of pohlianin C and analogues. *Bioorg. Med. Chem. Lett.* 24 2645–2647 10.1016/j.bmcl.2014.04.071 24813731

[B80] Lawyer C. Pai S. Watabe M. Borgia P. Mashimo T. Eagleton L. (1996). Antimicrobial activity of a 13 amino acid tryptophan-rich peptide derived from a putative porcine precursor protein of a novel family of antibacterial peptides. *FEBS Lett.* 390 95–98 10.1016/0014-5793(96)00637-0 8706838

[B81] Lax R. (2010). *The Future of Peptide Development in the Pharmaceutical Industry, Pharmanuacturing: the International Peptide Review*. Available at: http://www.polypeptide.com/web/upload/medias/1401702726538c49464a6f5.pdf (assessed November 21 2014).

[B82] Lax R. Meenan C. (2012a). *Challenges for Therapeutic Peptides Part 1: On the Inside, Looking Out, Innovations in Pharmaceutical Technology*. Available at: http://www.polypeptide.com/web/upload/medias/1401702387538c47f367ccc.pdf (assessed November 21 2014).

[B83] Lax R. Meenan C. (2012b). *Challenges for Therapeutic Peptides Part 2: Delivery Systems, Innovations in Pharmaceutical Technology*. Available at: http://www.polypeptide.com/web/upload/medias/1401702335538c47bf450c4.pdf (assessed November 21 2014).

[B84] Leavy O. (2010). Therapeutic antibodies: past, present and future. *Nat. Rev. Immunol.* 10 297 10.1038/nri2763 20422787

[B85] Leeson P. (2012). Drug discovery: chemical beauty contest. *Nature* 481 455–456 10.1038/481455a 22281594

[B86] Lehrer R. Barton A. Daher K. A. Harwig S. S. L. Ganz T. Selsted M. E. (1989). Interaction of human defensins with *Escherichia coli*. *J. Clin. Invest.* 84 553–561 10.1172/JCI114198 2668334 PMC548915

[B87] Lehrer R. I. Cole A. M. Selsted M. E. (2012). θ-defensins: cyclic peptides with endless potential. *J. Biol. Chem.* 287 27014–27019 10.1074/jbc.R112.346098 22700960 PMC3411038

[B88] Li W. F. Ma G. X. Zhou X. X. (2006). Apidaecin-type peptides: biodiversity, structure-function relationships and mode of action. *Peptides* 27 2350–2359 10.1016/j.peptides.2006.03.016 16675061

[B89] Lipinski C. A. Lombardo F. Dominy B. W. Feeney P. J. (1997). Experimental and computational approaches to estimate solubility and permeability in drug discovery and development settings. *Adv. Drug Deliv. Rev.* 23 3–25 10.1016/S0169-409X(96)00423-1 11259830

[B90] López-Rojas R. Docobo-Pérez F. Pachón-Ibáñez M. De La Torre B. G. Fernández-Reyes M. March C. (2011). Efficacy of cecropin A-melittin peptides on a sepsis model of infection by pan-resistant *Acinetobacter baumannii*. *Eur. J. Clin. Microbiol. Infect. Dis.* 11 1391–1398 10.1007/s10096-011-1233-y 21479973

[B91] Lowenberger C. (2001). Innate immune response of *Aedes aegypti*. *Insect Biochem. Mol. Biol.* 31 219–229 10.1016/S0965-1748(00)00141-7 11167091

[B92] Lowenberger C. Bulet P. Charlet M. Hetru C. Hodgeman B. Christensen B. M. (1995). Insect immunity: isolation of 3 novel inducible antibacterial defensins from the vector mosquito, *Aedes aegypti*. *Insect Biochem. Mol.* 25 867–873 10.1016/0965-1748(95)00043-U 7633471

[B93] Lowenberger C. Charlet M. Vizioli J. Kamal S. Richman A. Christensen B. M. (1999). Antimicrobial activity spectrum, cDNA cloning, and mRNA expression of a newly isolated member of the cecropin family from the mosquito vector *Aedes aegypti*. *J. Biol. Chem.* 274 20092–20097 10.1074/jbc.274.29.20092 10400619

[B94] Ludtke S. J. He K. Heller W. T. Harroun T. A. Yang L. Huang H. W. (1996). Membrane pores induced by magainin. *Biochemistry* 35 13723–13728 10.1021/bi9620621 8901513

[B95] Maia F. R. Barbosa M. Gomes D. B. Vale N. Granja P. Gomes P. (2014). Hydrogel depots for local co-delivery of osteoinductive peptides and mesenchymal stem cells. *J. Control. Release* 189 158–168 10.1016/j.jconrel.2014.06.030 24979208

[B96] Malpede B. M. Tolia N. H. (2014). Malaria adhesins: structure and function. *Cell. Microbiol.* 16 621–631 10.1111/cmi.12276 24506585 PMC4002501

[B97] Mantel P.-Y. Hoang A. N. Goldowitz I. Potashnikova D. Hamza B. Vorobjev I. (2013). Malaria-infected erythrocyte-derived microvesicles mediate cellular communication within the parasite population and with the host immune system. *Cell Host Microbe* 13 521–534 10.1016/j.chom.2013.04.009 23684304 PMC3687518

[B98] Mason A. J. Moussaoui W. Abdelrahman T. Boukhari A. Bertani P. Marquette A. (2009). Structural determinants of antimicrobial and antiplasmodial activity and selectivity in histidine-rich amphipathic cationic peptides. *J. Biol. Chem.* 284 119–133 10.1074/jbc.M806201200 18984589

[B99] Matsuda S. Koyasu S. (2000). Mechanisms of action of cyclosporin. *Immunopharmacology* 47 119–125 10.1016/S0162-3109(00)00192-2 10878286

[B100] McConkey G. A. Rogers M. J. McCutchan T. F. (1997). Inhibition of *Plasmodium falciparum* protein synthesis. Targeting the plastid like organelle with thiostrepton. *J. Biol. Chem.* 272 2046–2049 10.1074/jbc.272.4.2046 8999899

[B101] Melo M. N. Ferre R. Castanho M. A. (2009). Antimicrobial peptides: linking partition, activity and high membrane-bound concentrations. *Nat. Rev. Microbiol.* 7 245–250 10.1038/nrmicro2095 19219054

[B102] Nickell S. P. Scheibel L. W. Cole G. A. (1982). Inhibition by cyclosporin A of rodent malaria in vivo and human malaria in vitro. *Infect. Immun.* 37 1093–1100. 6752020 10.1128/iai.37.3.1093-1100.1982 PMC347653

[B103] Otvos L. Jr. (2005). Antibacterial peptides and proteins with multiple cellular targets. *J. Pept. Sci.* 11 697–706 10.1002/psc.698 16059966

[B104] Pelegrini P. Sarto R. Silva O. Franco O. L. Grossi-de-Sa M. F. (2011). Antibacterial peptides from plants: what they are and how they probably work. *Biochem. Res. Int.* 2011 1–9 10.1155/2011/250349 PMC3049328 21403856

[B105] Philips R. S. (2001). Current status of malaria and potential for control. *Clin. Microbiol. Rev.* 14 208–226 10.1128/CMR.14.1.208-226.2001 11148010 PMC88970

[B106] Portmann C. Sieber S. Wirthensohn S. Blom J. F. Da Silva L. Baudat E. (2014). Balgacyclamides, antiplasmodial heterocyclic peptides from *Microcystis aeruguinosa* EAWAG 251. *J. Nat. Prod.* 77 557–562 10.1021/np400814w 24392715

[B107] Possani L. D. Corona M. Zurita M. Rodríguez M. H. (2002). From noxiustoxin to scorpine and possible transgenic mosquitoes resistant to malaria. *Arch. Med. Res.* 33 398–404 10.1016/S0188-4409(02)00370-3 12234530

[B108] Pouny Y. Rapaport D. Mor A. Nicolas P. Shai Y. (1992). Interaction of antimicrobial dermaseptin and its fluorescently labeled analogues with phospholipid membranes. *Biochemistry* 31 12416–12423 10.1021/bi00164a017 1463728

[B109] Powers J.-P. S. Hancock R. E. W. (2003). The relationship between peptide structure and antibacterial activity. *Peptides* 24 1681–1691 10.1016/j.peptides.2003.08.023 15019199

[B110] Pretzel J. Mohring F. Rahlfs S. Becker K. (2013). Antiparasitic peptides. *Adv. Biochem. Eng. Biotechnol.* 135 157–192 10.1007/10_2013_191 23615879

[B111] Prudêncio M. Rodriguez A. Mota M. M. (2006). The silent path to thousands of merozoites: the *Plasmodium* liver stage. *Nat. Rev. Microbiol.* 4 849–856 10.1038/nrmicro1529 17041632

[B112] Pushpanathan M. Gunasekaran P. Rajendhran J. (2013). Antimicrobial peptides: versatile biological properties. *Int. J. Pept.* 2013:675391 10.1155/2013/675391 PMC3710626 23935642

[B113] Raghuraman H. Chattopadhyay A. (2007). Melittin: a membrane-active peptide with diverse functions. *Biosci. Rep.* 27 189–223 10.1007/s10540-006-9030-z 17139559

[B114] Regev-Rudzki N. Wilson D. W. Carvalho T. G. Sisquella X. Coleman B. M. Rug M. (2013). Cell-cell communication between malaria-infected red blood cells via exosome-like vesicles. *Cell* 153 1120–1133 10.1016/j.cell.2013.04.029 23683579

[B115] Robert A. Dechy-Cabaret O. Cazelles J. Meunier B. (2002). From mechanistic studies on artemisinin derivatives to new modular antimalarial drugs. *Acc. Chem. Res.* 35 167–174 10.1021/ar990164o 11900520

[B116] Rodriguez M. D. C. Zamudio F. Torres J. A. Gonzalez-Ceron L. Possani L. D. Rodriguez M. H. (1995). Effect of a cecropin-like synthetic ppetide (Shiva-3) on the sporogonic development of *Plasmodium berghei*. *Exp. Parasitol.* 80 596–604 10.1006/expr.1995.1075 7758540

[B117] Rotem S. Mor A. (2009). Antimicrobial peptide mimics for improved therapeutic properties. *Biochim. Biophys. Acta* 1788 1582–1592 10.1016/j.bbamem.2008.10.020 19028449

[B118] Schoof S. Pradel G. Aminake M. N. Ellinger B. Baumann S. Potowski M. (2010). Antiplasmodial thiostrepton derivatives: proteasome inhibitors with a dual mode of action. *Angew. Chem. Int. Ed. Engl.* 49 3317–3321 10.1002/anie.200906988 20358566

[B119] Selsted M. E. Harwig S. S. Ganz T. Schilling J. W. Lehrer R. I. (1985). Primary structures of three human neutrophil defensins. *J. Clin. Invest.* 76 1436–1439 10.1172/JCI112121 4056036 PMC424095

[B120] Sgolastra F. Deronde B. M. Sarapas J. M. Som A. Tew G. N. (2013). Designing mimics of membrane active proteins. *Acc. Chem. Res.* 46 2977–2987 10.1021/ar400066v 24007507 PMC4106261

[B121] Simmaco M. Mignogna G. Barra D. (1999). Antimicrobial peptides from amphibian skin: what do they tell us? *Biopolymers* 47 435–450 10.1002/(SICI)1097-0282(1998)47:6<435::AID-BIP3>3.0.CO;2-8 10333736

[B122] Singh B. Daneshvar C. (2013). Human infections and detection of *Plasmodium knowlesi*. *Clin. Microbiol. Rev.* 26 165–184 10.1128/CMR.00079-12 23554413 PMC3623376

[B123] Som A. Vemparala S. Ivanov I. Tew G. N. (2008). Synthetic mimics of antimicrobial peptides. *Biopolymers* 90 83–92 10.1002/bip.20970 18314892

[B124] Sorensen O. Cowland J. B. Askaa T. Borregaard N. (1997). An ELISA for hCAP-18, the cathelicidin present in human neutrophils and plasma. *J. Immunol. Methods* 206 53–59 10.1016/S0022-1759(97)00084-7 9328568

[B125] Sullivan D. J. Jr. Gluzman I. Y. Russell D. G. Goldberg D. E. (1996). On the molecular mechanism of chloroquine’s antimalarial action. *Proc. Natl. Acad. Sci. U.S.A.* 93 11865–11870 10.1073/pnas.93.21.11865 8876229 PMC38150

[B126] Tanaka H. Ishibashi J. Fujita K. Nakajima Y. Sagisaka A. Tomimoto K. (2008). A genome-wide analysis of genes and gene families involved in innate immunity of *Bombyx mori*. *Insect Biochem. Mol. Biol.* 38 1087–1110 10.1016/j.ibmb.2008.09.001 18835443

[B127] Teixeira C. Vale N. Pérez B. Gomes A. Gomes J. R. B. Gomes P. (2014). “Recycling” classical drugs for malaria. *Chem. Rev.* 114 11164–11220 10.1021/cr500123g 25329927

[B128] Tian C. Gao B. Rodriguez M. C. Lanz-Mendoza H. Ma B. Zhu S. (2008). Gene expression, antiparasitic activity, and functional evolution of the drosomycin family. *Mol. Immunol.* 45 3909–3916 10.1016/j.molimm.2008.06.025 18657321

[B129] Vale N. Moreira R. Gomes P. (2009). Primaquine revisited six decades after its discovery. *Eur. J. Med. Chem.* 44 937–953 10.1016/j.ejmech.2008.08.011 18930565

[B130] van Kan E. J. Demel R. A. Breukink E. van der Bent A. de Kruijff B. (2002). Clananin permeabilizes target membranes via two distinctly different pH-dependent mechanisms. *Biochemistry* 18 7529–7539 10.1021/bi012162t 12056883

[B131] Vijay S. Rawat M. Adak T. Dixit R. Nanda N. Srivastava H. (2011). Parasite killing in malaria non-vector mosquito *Anopheles culicifacies* species b: implication of nitric oxide synthase upregulation. *PLoS ONE* 6:e18400 10.1371/journal.pone.0018400 PMC3070730 21483693

[B132] Vila-Perelló M. Andreu D. (2005). Characterization and structural role of disulfide bonds in a highly knotted thionin from *Pyrularia pubera*. *Biopolymers* 80 697–707 10.1002/bip.20270 15765547

[B133] Visser B. J. van Vugt M. Grobusch M. P. (2014). Malaria: an update on current chemotherapy. *Expert Opin. Pharmacother.* 15 2219–1154 10.1517/14656566.2014.944499 25110058

[B134] Vizioli J. Bulet P. Charlet M. Lowenberger C. Blass C. Muller H. M. (2000). Cloning and analysis of a cecropin gene from the malaria vector mosquito, *Anopheles gambiae*. *Insect Mol. Biol.* 9 75–84 10.1046/j.1365-2583.2000.00164.x 10672074

[B135] Vizioli J. Bulet P. Hoffmann J. A. Kafatos F. C. Muller H. M. Dimopoulos G. (2001). Gambicin: a novel immune responsive antimicrobial peptide from the malaria vector *Anopheles gambiae*. *Proc. Natl. Acad. Sci. U.S.A.* 98 12630–12635 10.1073/pnas.221466798 11606751 PMC60105

[B136] Wang G. Li X. Wang Z. (2009). APD2: the updated antimicrobial peptide database and its application in peptide design. *Nucleic Acids Res.* 37 D933–D937 10.1093/nar/gkn823 18957441 PMC2686604

[B137] Wang S. Ghosh A. K. Bongio N. Stebbings K. A. Lampe D. J. Jacobs-Lorena M. (2012). Fighting malaria with engineered symbiotic bacteria from vector mosquitoes. *Proc. Natl. Acad. Sci. U.S.A.* 109 12734–12739 10.1073/pnas.1204158109 22802646 PMC3412027

[B138] Wang Z. Wang G. (2004). APD: The antimicrobial peptide database. *Nucleic Acids Res.* 32 D520–D592 10.1093/nar/gkh025 14681488 PMC308759

[B139] Wegscheid-Gerlach C. Gerber H. D. Diederich W. E. (2010). Proteases of *Plasmodium falciparum* as potential drug targets and inhibitors thereof. *Curr. Top. Med. Chem.* 10 346–367 10.2174/156802610790725461 20166950

[B140] Wei S.-Y. Wu J.-M. Kuo Y.-Y. Chen H.-L. Yip B.-S. Tzeng S.-R. (2006). Solution structure of a novel tryptophan-rich peptide with bidirectional antimicrobial activity. *J. Bacteriol.* 188 328–334 10.1128/JB.188.1.328-334.2006 16352849 PMC1317575

[B141] Wells T. M. Poll E. M. (2010). When is enough enough? The need for a robust pipeline of high-quality antimalarials. *Discov. Med.* 9 389–398. 20515606

[B142] White N. J. Pukrittayakamee S. Hien T. T. Faiz M. A. Mokuolu O. A. Dondorp A. M. (2014). Malaria. *Lancet* 383 723–735 10.1016/S0140-6736(13)60024-0 23953767

[B143] WHO. (2012). *World Malaria Report.* Geneva: World Health Organization.

[B144] Wieczorek M. Jenssen H. Kindrachuk J. Scott W. R. P. Elliott M. Hilpert K. (2010). Structural studies of a peptide with immune modulating and direct antimicrobial activity. *Chem. Biol.* 17 970–980 10.1016/j.chembiol.2010.07.007 20851346

[B145] Wimley W. C. Selsted M. E. White S. H. (1994). Interactions between human defensins and lipid bilayers: evidence for formation of multimeric pores. *Protein Sci.* 3 1362–1373 10.1002/pro.5560030902 7833799 PMC2142938

[B146] Yang L. Harroun T. A. Weiss T. M. Ding L. Huang H. W. (2001). Barrel-stave model or toroidal model? A case study on melittin pores. *Biophys. J.* 81 1475–1485 10.1016/S0006-3495(01)75802-X 11509361 PMC1301626

[B147] Yeaman M. R. Yount N. Y. (2003). Mechanisms of antimicrobial peptide action and resistance. *Pharmacol. Rev.* 55 27–55 10.1124/pr.55.1.2 12615953

[B148] Yi H. Y. Chowdhury M. Huang Y. D. Yu X. Q. (2014). Insect antimicrobial peptides and their applications. *Appl. Microbiol. Biotechnol.* 98 5807–5822 10.1007/s00253-014-5792-6 24811407 PMC4083081

[B149] Zasloff M. (1987). Magainins, a class of antimicrobial peptides from *Xenopus* skin: isolation, characterization of two active forms, and partial cDNA sequence of a precursor. *Proc. Natl. Acad. Sci. U.S.A.* 84 5449–5453 10.1073/pnas.84.15.5449 3299384 PMC298875

[B150] Zasloff M. (2002). Antimicrobial peptides of multicellular organisms. *Nature* 415 389–395 10.1038/415389a 11807545

